# MTHFR Gene Polymorphism and Age of Onset of Schizophrenia and Bipolar Disorder

**DOI:** 10.1155/2014/318483

**Published:** 2014-07-03

**Authors:** Mohamed A. El-Hadidy, Hanaa M. Abdeen, Sherin M. Abd El-Aziz, Mohammad Al-Harrass

**Affiliations:** ^1^Psychiatric Department, Faculty of Medicine, Mansoura University, 45 El Thorah Street, El-Refaay Tower, Mansoura 35111, Egypt; ^2^Medical Biochemistry Department, Faculty of Medicine, Mansoura University, Mansoura 35111, Egypt; ^3^Clinical Pathology Department, Faculty of Medicine, Mansoura University, Mansoura 35111, Egypt

## Abstract

*Objective*. Several studies with contradictory results from different cultures about association of methylenetetrahydrofolate reductase (MTHFR) C677T polymorphism in schizophrenia and bipolar disorders. Little is known about this association in Arab culture and Egypt. So the present study aimed to assess the association of MTHFR C677T polymorphism in bipolar disorder (BD) and schizophrenia in comparison to control group. The association between MTHFR C677T polymorphism and the age at onset in schizophrenia or BD was also studied. *Methods*. Polymerase chain reaction and restriction fragment length polymorphism (PCR-RFLP) were used to examine the genotype and allele frequencies of MTHFR C677T polymorphism in 149 healthy subjects and 134 bipolar and 103 schizophrenia patients. *Results*. In BD and schizophrenia, there was a higher prevalence of MTHFR C677T polymorphism than healthy subjects. Earlier age at onset was found in patients with BD, carrying one copy of the T allele or CT genotypes but not in patients with schizophrenia. *Conclusion*. The present findings suggest that the MTHFR C677T polymorphisms are likely to be associated with the risk of developing BD and schizophrenia and influence the age at onset of BD but not the age at onset of schizophrenia.

## 1. Introduction

Schizophrenia and bipolar disorder (BD) are functional psychiatric disorders that have been linked to low folate levels and defective folate metabolism [1, 2]. Schizophrenia is a common serious psychotic illness, with a lifetime incidence of 1-2% [3]. It is characterized by disorders of thought and volition and by abnormal perceptions (hallucinations) and beliefs (delusions) [4]. On the other hand, BD is one of major psychiatric disorders with rigorous lifelong disability and has a lifetime prevalence of 0.3–1.6%, with equal prevalence among men and women [5, 6]. It is characterized by severe alteration in mood, with periods of depression and mania or, less commonly, mania only. Both disorders are a complex disorder involving multiple genetic and environmental factors [7, 8]. A lot of genetic loci contributing to schizophrenia and bipolar disorders have been identified in recent years through genome-wide association studies [9–12]. The methylenetetrahydrofolate reductase (MTHFR) gene polymorphism could be one of the risk factors of overall schizophrenia and BD [13–15].

MTHFR gene is located at the end of the short arm of chromosome 1 (1p36.3). The enzyme plays a central role in folate metabolism by irreversibly converting 5, 10-methylenetetrahydrofolate (5, 10-MTHF) to 5-MTHF, the predominant circulating form of folate [16]. 5-MTHF plays a crucial role in one-carbon metabolism and DNA methylation [17]. It donates a methyl group to homocysteine in the generation of S-adenosylmethionine, a major source of methyl groups in the brain [16]. In addition to this, homocysteine and its metabolites may have a direct excitotoxic effect on the N-methyl-D-aspartate (NMDA) glutamate receptors in the brain and may inhibit methylation processes in the central nervous system [18]; therefore, its conversion is critical [19]. So normal activity of this enzyme maintains the pool of circulating folate and methionine and reduces levels of homocysteine [20].

Common single nucleotide polymorphisms in MTHFR have been reported, a C/T transition at nucleotide 677 in exon 4 (rs1801133) [21] that causes an alanine to valine (Al222Val) amino acid substitution [19]. This polymorphism is functional and results in diminished enzyme activity [20]. This single amino acid substitution results in impaired flavin adenine dinucleotide (FAD) binding, leading to loss of folate resulting, in its turn, in reduced activity of MTHFR [22]. For the C677T polymorphism, homozygote variants have 30% enzyme activity in comparison with homozygote for the wild-type C allele, while heterozygote retains 65% of wild-type MTHFR enzyme activity [20].

Age at onset in schizophrenia is variable and may occur in early childhood or late in life [23] and has been found to have genetic components [21, 24]. Age at onset has been regarded as a proxy measure of disease severity. An early age at onset has been associated with a range of more severe deficiencies in areas such as cognition, clinical and behavioral presentation, social ability, and brain ventricle sizes [21]. In General, age at onset has clinical and prognostic significance in psychotic disorders and may be under considerable genetic control [24, 25].

The aim of this work was to investigate the possible role of the MTHFR C677T polymorphism in the risk of developing BD and schizophrenia and also to study the association between this polymorphism and the age at onset in both psychiatric disorders.

## 2. Methods

First 6-month duration of this study was spent to select suitable patients who could participate in this study by examining all patients who came to the Department of Psychiatry (inpatient and outpatients clinics) attached to Mansoura University Hospital for any cause (new cases, follow-up cases, and cases came for admission). At the end of this 6-month study, 621 patients fulfill the Diagnostic and Statistical Manual 4th edition—Text Revision (DSM IV-TR) criteria for either of schizophrenia or mood disorder bipolar I [26]. All patients were interviewed using the Mini-International Neuropsychiatric Interview (MINI). MINI is a short structured diagnostic interview. The scale had been previously translated into Arabic and validated [27]. The diagnosis was further validated by at least two consultants of psychiatry who had 10-year clinical experience.

At the end of this survey and after explaining to the patients and their relatives the aims of this study, 186 patients refuse to participate in the study or refuse to give a blood sample. Also 165 patients were not included in the study because they met one of the exclusion criteria. Exclusion criteria were subjects with a history of brain injury; neurological disorders; psychiatric disorders due to general medical conditions or substance abuse; or subjects related to an individual already included in the study. Also 33 patients with some chronic medical diseases or known genetic disease were excluded. Finally, 237 patients completed this study (103 patients diagnosed with schizophrenia and 134 patients diagnosed with BD). The clinical data were summarized in Table 1.

149 healthy volunteers (control group) were randomly recruited from relatives of other patients in the Medicine Department, outpatient clinic, in the same hospital, with negative family or past history of any psychiatric disorders and with no family relationship to the present study patients. A written informed consent was obtained from all participants. The study was approved by the local ethics committee. The study has been performed in accordance with the ethical standards laid down in the 1964 Declaration of Helsinki.

### 2.1. DNA Extraction and MTHFR C677T Genotyping

From all participants, 5 mL venous blood samples were obtained on EDTA containing tubes. Genomic DNA was isolated from peripheral blood leukocyte using the Gentra Puregene Blood Kit (Qiagen Inc., Valencia, CA, USA). The extracted DNA was stored at 4°C until analysis.

Genotyping of the MTHFR C677T was performed by polymerase chain reaction and restriction fragment length polymorphism (PCR-RFLP) [28, 29]. PCR amplification was performed using 5′-CAAAGGCCACCCCGAAGC 3′ and 5′-AGGACGGTGCGGTGAGAGTG-3′ as the forward and reverse primer pairs, respectively. Each amplification reaction was performed in a total volume of 25 *μ*L, containing 1.8 mM MgCl2, 1 U Taq polymerase, 2.5 mmol/L of each dNTP, 5 pmol/L of each primer, and 100 ng of genomic DNA, processing starts with 94°C for 5 min and 34 cycles at 94°C for 45 s, 62°C for 40 s, and 72°C for 50 s. This was followed by a final extension at 72°C for 7 min. Then, 5 U of FastDigest HinfI (Fermentas International Inc.) was added to the PCR products (5 *μ*L) and digested at 37°C for 30 minutes. After restriction enzyme digestion of the amplified DNA, genotypes were identified by electrophoresis on 3% agarose gels and visualized with ethidium-bromide staining ultraviolet illumination.

### 2.2. Electrophoresis and Genotyping

After the genomic DNA of the samples was amplified by PCR and imaged, the purpose gene of 245 bp nucleotide sequences was studied. The genotypes identified were named according to the presence or absence of the enzyme restriction sites, when a C to T transversion at nucleotide position 677 of the MTHFR gene occurs. The presence of the cutting site indicates the T allele, while its absence indicates the C allele. Thus, the CC genotype is homozygote for the absence of the site (band at 245 bp), CT genotype is heterozygote for the absence and presence of the site (bands at 245, 173, and 72 bp), and TT genotype is homozygote for the presence of the site (bands at 173 and 72 bp).

### 2.3. Statistical Analysis

All statistical analyses were done with the SPSS 20.0 package (Statistical Package for the Social Sciences, SPSS Inc., Chicago, Illinois, USA). Quantitative data were summarized using mean and stander deviation (SD). The statistical significant difference between patient and control groups was tested using *t*-test and ANOVA test. Statistical significance was defined as *P* < 0.05. Qualitative variables were expressed as number and percentages. The allele frequencies in the groups of affected subjects and controls were compared using a Chi-square statistic with 1 degree of freedom. The Hardy-Weinberg test was done to examine whether the allele and genotype frequencies in the studied groups remain constant from generation to generation in the absence of other evolutionary influences or not. Linear regression analysis was done for age of onset as the dependent variable and allele frequency as a predictor factor.

## 3. Results

**Figure 1:**
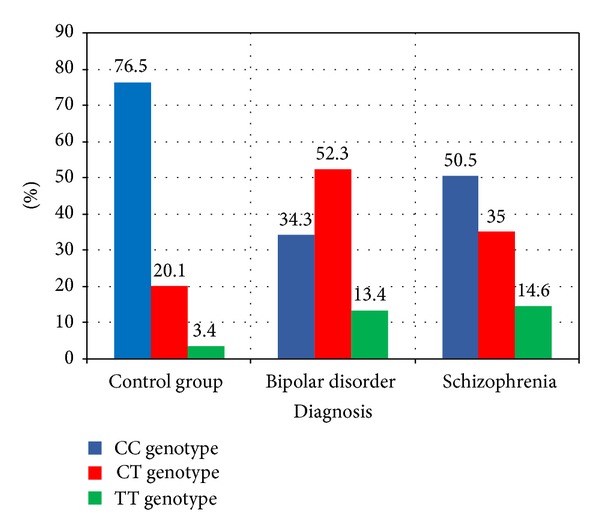
It shows the genotype distribution among the studied groups.

**Figure 2:**
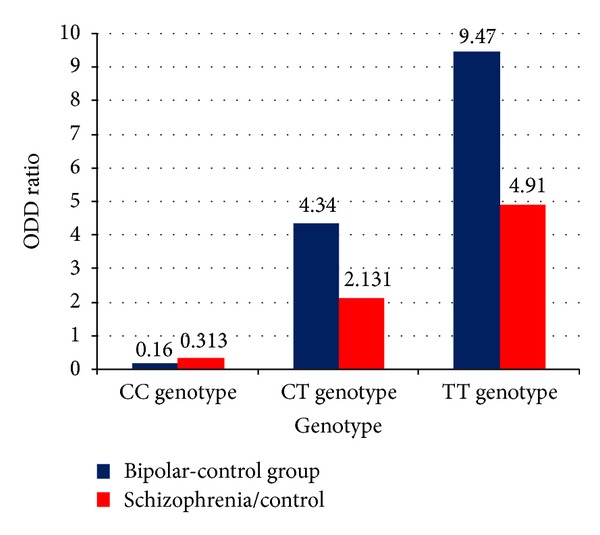
It shows the odds ratio of genotype in the studied groups.

Table 2 shows that the Hardy-Weinberg test is insignificant in all studied groups which mean that allele and genotype frequencies remain constant from generation to generation in the absence of other evolutionary influences. It also shows that there is a significant increase in CC genotype distribution in the control group when compared to both BD and schizophrenia groups (OR (CI) = 0.16 (0.095–0.27), *P* ≤ 0.000, and OR (CI) = 0.313 (0.182–0.538), *P* = 0.000, resp.), while the CT genotype distribution is significantly increased in BD and schizophrenia groups versus control group (OR (CI) = 4.34 (2.57–7.33), *P*≤0.001 and 2.131 (1.21–3.77) *P* = 0.009, resp.). Also, the TT genotype distribution is significantly increased in BD and schizophrenia groups versus control group (OR (CI) = 9.47 (1.61–12.4), *P* = 0.002 and 4.91 (1.72–13.98) *P* = 0.001, resp.). The C allele is significantly prevalent in the control group compared to the BD and schizophrenia groups (OR (CI) = 0.17 (0.08–0.36), ≤0.001, and OR (CI) = 0.18 (0.09–0.38), *P* ≤ 0.001, resp.), while T allele is prevalent in BD patients and schizophrenia patients compared to the control group with a statically significant difference (OR (CI) = 6.57 (4.24–10.17), *P* < 0.001, and OR (CI) = 3.62 (2.29–5.71) *P* < 0.001, resp.)(Figures 1 and 2).

From Table 3, there is a significant decrease in mean age at onset found for MTHFR C677T polymorphism in BD patients, in patients with CT genotype, compared to CC genotype (*P* = 0.004). For schizophrenia patients, there is a significant decrease in mean age at onset with the TT genotype compared to CT genotype (*P* = 0.046). The presence of one copy of the wild C allele showed older age at onset in BD patients (*P* = 0.032) and the presence of one copy of T allele is associated with earlier age of onset in BD (*P*≤0.001). On the other hand, in schizophrenia, none of T or C allele was associated with a significant difference in mean age at onset (*P* = 0.66 and *P* = 0.10). In addition, Table 4 shows that the T allele is a good predictor for early onset of bipolar disorders but not in schizophrenia.

## 4. Discussion

The elucidation of etiology and pathophysiology of the disease is extremely important to establish treatment and prevention strategies [30]. Schizophrenia and BD are psychiatric disorders that involve multiple genetic and environmental factors. They have been linked to low folate and defective folate metabolism where MTHFR enzyme plays a central role [31].

The present study showed an association of MTHFR C677-T polymorphism with BD as CT, TT genotypes and T allele is more prevalent in BD patients. This result is in part in accordance with Kempisty et al. [32] that indicate a possible association of BD (type 1) with the 1p36.3 MTHFR (C677T) locus. Also, Gilbody et al. [31] and Jönsson et al. [33] in their meta-analysis on BD reported almost significant or borderline significant association with MTHFR C677T, and the frequency of the T allele was increased among BD patients [31, 33]. Also, the meta-analysis conducted by Peerbooms et al. [34] indicated that carriers of the T allele and TT genotype are at a small statistically significant increasing the risk of the major psychiatric disorders including schizophrenia and BD [34].

On the other hand, Tan et al. [35] found that here was no significant difference in genotype distributions or allele frequencies between controls and any of the diagnostic groups they have studied (schizophrenia and BD), although the frequency of the T allele was higher in these two groups. As there was no significant association as measured by the *P* value, the odds ratio and confidence interval provided some evidence of increased risk for individuals with the T-containing genotypes. And the role of this polymorphism in the pathogenesis of schizophrenia and BD could not be ruled out [35]. Chen et al. [36] also found no significant difference in either allele frequencies or genotype distribution between BD patients and controls in their association study in the Chinese population and also in the meta-analysis they have conducted [36].

Moreover the meta-analyses conducted by Zintzaras [37] and Cohen-Woods et al. [19] found that there was no significant association between either allele of the C677T polymorphism and the risk of developing BD [19, 37]. These discrepancies, in part, may result from hidden population stratifications, explicitly, socioeconomic status. Convincingly, dietary folate content has effects on enzyme activity of MTHFR that indicate possible compensatory effects of folate on defective enzyme activity [17].

The current study also showed an association between MTHFR C677-T polymorphism and schizophrenia as TT genotype and T allele are more prevalent in schizophrenia patients. This result is in harmony with that of Zintzaras [37] who found that the TT genotype of the MTHFR C677T polymorphism contributes to the susceptibility of schizophrenia. Also Kempisty et al. [32] indicate a possible association of schizophrenia with the 1p36.3 MTHFR (C677T) locus [32]. Moreover, meta-analyses studies conducted by Lewis et al. [38], Muntjewerff et al. [39], Gilbody et al. [31], Yoshimi et al. [40], and Peerbooms et al. [34], tested for an association between MTHFR C677T and schizophrenia, reported an increased risk of schizophrenia with the TT genotype and the carriers of the T allele.

Also, this result is partially in accordance with that of Lajin et al. [41] who found a statistical significant association for MTHFR 677TT genotype under the recessive model in the male patients subgroup and MTHFR 677CT genotype under the over dominant model in the total patient group [41]. On the other hand, Kim et al. [42] found that there was no significant association between MTHFR C677T polymorphism and the risk of schizophrenia, similar to the results obtained by Tan et al. [35] and Kim et al. [42]. But they meta-analyzed the previous case-control studies for the C677T by ethnic groups and showed a significant association in the combined and Asian populations but not in the Korean and Caucasian populations alone [42]. This controversy may be explained by the demographic differences, such as ethnicity, that may influence the association of the MTHFR polymorphisms with psychiatric disorders [37, 42].

MTHFR is a crucial enzyme involved in one-carbon metabolism, which is a folate-mediated pathway [43]. It is a complex pathway and regulates not only nucleotide synthesis, but also DNA methylation [16]. MTHFR catalyses the conversion of 5, 10-MTHF to 5-MTHF, the predominant circulating form of folate. 5, 10-MTHF is involved in DNA synthesis as an essential donor molecule for purine synthesis and a substrate molecule for thymidine syntheses, which is a rate limiting step in DNA biosynthesis. Also, the methyl group of 5-MTHF is furthermore used for the remethylation of homocysteine to methionine and the conversion of methionine to S-adenosylmethionine (SAM), which is a major methyl donor to DNA, proteins, neurotransmitters, hormones, and phospholipids [34, 43, 44]. SAM is a major source of methyl groups in the brain [31]. The MTHFR C677T polymorphism is associated with a reduction in the bioavailability of folate and folate metabolites and “mimics” low dietary folate intake. Therefore, MTHFR polymorphisms can increase serum homocysteine level and as a consequence result in an impairment of DNA methylation. Both of these are known to be risk factors for schizophrenia and other neuropsychiatric disorders [40].

DNA methylation is a critical epigenetic modification of the genome that controls many biologic processes, including embryonic development, X chromosome inactivation, imprinting, and gene expression [45, 46]. Methylation is genetically predetermined, either by imprinting or by inheritance of genes which influence methylation, such as MTHFR and other genes involved in the one-carbon cycle [47]. Methyl groups required for methylation are synthesized de novo or are supplied in the diet, primarily from folate. Thus, methylation may be modified by gene-exposure interactions occurring during development. Incorrect methylation patterns can affect embryogenesis, leading to developmental malformations and embryonic death [31]. Although methylation patterns are established during early life, they are not fixed, and gradual hypomethylation of the genome occurs in most tissues with age, together with aberrant hypermethylation of gene promoter regions. Thus, the correct establishment of DNA methylation patterns is important not only during early life, but also for long-term health benefits, including psychiatric and neurologic disease susceptibility [48].

Schizophrenia is increasingly considered to be a neurodevelopmental disorder, with in utero exposures and epigenetic mechanisms such as DNA methylation being important in its etiology [31]. Besides, schizophrenia and BD are the result of a complex gene-environmental implication whose epigenetic factors have been proposed for importance [49].

The absence of clear evidence of major genetic effects, despite (high) estimates of heritability for major psychiatric disorders, together with evidence of “causal” environmental exposures resulting in changes in gene expression is consistent with the concept that the biologic underpinnings of psychiatric disorders are epigenetic in form rather than DNA sequence based [50]. This forms a plausible biologic explanation for potential associations between genetic variation in folate metabolism and schizophrenia and other functional psychiatric disorders [31].

This study also showed an association between MTHFR C677T polymorphism in genotype CT and T allele frequencies with earlier age at onset of BD. To our knowledge, no study examines age of onset in BD and its association with MTHFR C677T polymorphism. But for schizophrenia, in this study, there was no association between MTHFR C677T polymorphism in genotype and allele frequencies with the age at onset. This result is in harmony with studies by Peerbooms et al. [25] and Saetre et al. [21] showing no large or significant differences in age at onset of schizophrenia for MTHFR C677T [21, 25]. Also, Saetre et al. [51] found no significant association between the MTHFR C677T polymorphism and age at onset of schizophrenia in the Nordic population [51]. However, the study conducted by Vares et al. [52] showed significant association between the MTHFR C677T polymorphism and age at onset in schizophrenia, as the mutant T allele was associated with earlier age at onset in unrelated schizophrenia patients from three Scandinavian samples and in Chinese high-risk families with multiple affected siblings [52].

The observed heterogeneity in the associations between MTHFR polymorphisms and age of onset between studies may reflect heterogeneity in the assessed trait and gene-environment and/or gene-gene interactions [51]. Also, it may reflect the dissimilarities between samples, with different ethnicities, ascertainment routes, definition of age at onset, gender and different genotype assays (where the more recent technology used may eliminate some of the possible errors obtained). Also, we cannot rule out that the difference in estimated response to the C677T genotypes may be due to chance alone [52].

One limitation of this study is the unavailability of studies in Arab countries examining this gene and any other genetic studies in general. This makes comparison with previous studies from Arab countries so difficult, so we compare the present results with results in other (Caucasian) populations.

In conclusion, the present study provides evidence that the MTHFR C677T variants may influence the risk of developing BD and schizophrenia. Furthermore, the polymorphism may influence the age at onset of BD but not the age at onset of schizophrenia. Additional genetic studies are needed to further analyze this topic.

## Figures and Tables

**Table 1:** Clinical data of the control, bipolar, and schizophrenia groups.

	Control (149)	Bipolar (134)	Schizophrenia (103)	Significance
Sex				
Female (%)	73 (49.0%)	62 (46.3%)	35 (34%)	*X* ^2^ = 5.983 *P* = 0.051
Male (%)	76 (51.0%)	72 (53.7%)	68 (66%)	
Residence				
Urban	73 (49%)	64 (47.8%)	55 (53.4%)	*X* ^2^ = 0.794 *P* = 0.672
Rural	76 (51%)	70 (52.2%)	48 (46.6%)	
Family History				
Negative	149 (100%)	78 (58.2%)	67 (65%)	*X* ^2^ = 77.44 *P* = 0.000
Positive	—	56 (41.8%)	36 (35%)	
Age (Years ± SD)	34.3 ± 6.0	32.2 ± 10.9	33.9 ± 9.4	*F* = 0.015 *P* = 0.903
Age of onset at diagnosis (Years ± SD)	—	20.4 ± 8.3	20.25 ± 6.4	*t* = 0.122 *P* = 0.9

Data are numbered (%) and mean ± SD.

**Table 2:** Genotype and allele frequencies of the C677T SNP in the control, bipolar, and schizophrenia groups.

	Control	Bipolar	Schizophrenia	Bipolar-control			Schizophrenia-control		
	(*N* = 149)	(*N* = 134)	(*n* = 103)	OR (CI)	Pearson *x* ^2^ (df = 2)	*P* value	OR (CI)	Pearson *x* ^2^ (df = 2)	*P* value
Genotype:									
CC	114 (76.5%)	46 (34.3%)	52 (50.5%)	0.16 (0.095–0.27)	51.1	0.000∗	0.313 (0.182–0.538)	18.35	0.000∗
CT	30 (20.1%)	70 (52.3%)	36 (35%)	4.34 (2.57–7.33)	31.8	0.000∗	2.131 (1.21–3.77)	6.917	0.009
TT	5 (3.4%)	18 (13.4%)	15 (14.6%)	9.47 (1.61–12.4)	9.6	0.002	4.91 (1.72–13.98)	10.47	0.001
Hardy-Weinberg equilibrium	*X* ^2^ = **2.66** *P* = 0.103	*X* ^2^ = **1.15** *P* = 0.2844	*X* ^2^ = **0.91** *P* = 0.339						

Allele frequencies:									
C	258 (96.3%)	162 (81.8%)	140 (82.4%)	0.17 (0.08–0.36)	26.73	0.000	0.18 (0.09–0.38)	24.3	0.000
T	40 (14.9%)	106 (53.5%)	66 (38.8%)	6.57 (4.24–10.17)	78.9	0.000∗	3.62 (2.29–5.71)	32.4	0.000∗

*Significance (*P* < 0.05). OR: odd ratio. CI: confidence interval.

**Table 3:** Mean age at onset and results of ANOVA test for genotype and allele frequency of MTHFR C677T in the bipolar and schizophrenia groups.

Diagnosis	Genotype	Age at onset (Years)	*F*	*P* value	Scheffe test (*P* value)			Allele frequencies		Age at onset (Years)	*t*	*P* value
		Mean ± SD			CC-CT	CC-TT	CT-TT			Mean ± SD	
Bipolar disorder (*N* = 134)	CC	23.65 ± 7.99	5.876	0.004	0.004	0.145	0.946	C	Present	21.43 ± 8.6	2.176	0.032
	CT	18.51 ± 8.7							Absent	19.22 ± 4.5		
	TT	19.22 ± 4.6						T	Present Absent	18.75 ± 7.5 23.65 ± 7.94	−4.445	0.000

Schizophrenia (*N* = 136)	CC	19.96 ± 5.37	10.74	0.000∗	−0.06	0.01	0.000	C	Present	20.74 ± 6.09	5.42	0.00
	CT	23.00 ± 7.5							Absent	14.67 ± 1.5		
	TT	14.67 ± 1.5						T	Present Absent	19.21 ± 7.0 19.96 ± 5.4	0.79	0.431

SD: standard deviation. ∗Significance (*P* < 0.05).

**Table 4:** Regression analysis for age of onset and allele frequency.

Diagnosis	Model		Unstandardized coefficients		Standardized coefficients	*t*	*P*
			B	Std. Error	Beta	
Schizophrenia	1	(Constant)	11.628	1.451		1.015	0.312
		C	8.333	1.349	0.029	1.179	0.240
		T	3.038	1.055	0.147	1.880	0.061

Bipolar disorder	1	(Constant)	24.360	1.782		13.666	0.000
		C	−0.708	1.589	−0.034	−0.446	0.656
		T	−5.138	1.229	−0.317	−4.181	0.000

(a) Dependent variable: age of onset.
